# Underdetermined Blind Source Separation of Synchronous Orthogonal Frequency Hopping Signals Based on Single Source Points Detection

**DOI:** 10.3390/s17092074

**Published:** 2017-09-11

**Authors:** Chaozhu Zhang, Yu Wang, Fulong Jing

**Affiliations:** College of Information and Communication Engineering, Harbin Engineering University, Harbin 150001, China; zhangchangzhu@hrbeu.edu.cn (C.Z.); jing_fl@hrbeu.edu.cn (F.J.)

**Keywords:** underdetermined blind source separation, frequency hopping sgnals, direction-of-arrival, mixing matrix estimation

## Abstract

This paper considers the complex-valued mixing matrix estimation and direction-of-arrival (DOA) estimation of synchronous orthogonal frequency hopping (FH) signals in the underdetermined blind source separation (UBSS). A novel mixing matrix estimation algorithm is proposed by detecting single source points (SSPs) where only one source contributes its power. Firstly, the proposed algorithm distinguishes the SSPs by the comparison of the normalized coefficients of time frequency (TF) points, which is more effective than existing detection algorithms. Then, mixing matrix of FH signals can be estimated by the hierarchical clustering method. To sort synchronous orthogonal FH signals, a modified subspace projection method is presented to obtain the DOAs of FH. One superiority of this paper is that the estimation accuracy of the mixing matrix can be significantly improved by the proposed SSPs detection criteria. Another superiority of this paper is that synchronous orthogonal FH signals can be sorted in underdetermined condition. The experimental results demonstrate the efficiency of the two proposed algorithms.

## 1. Introduction

Frequency hopping (FH) signals have been widely used in wireless communications and radar systems due to their low probability of detection and interception [1,2,3,4,5]. To obtain useful information in communication reconnaissance field, it is an important task to sort multiple FH signals. Hop timing, frequency, and direction of arrival (DOA) are three important physical quantities for sorting FH signals. DOA is a vital parameter in radar and wireless communication applications [6,7,8,9,10,11,12,13,14]. Many methods for estimating FH parameters have been reported [15,16,17,18]. However, the methods in [15,16,17,18] can only estimate asynchronous FH signal parameters under overdetermined condition, in which the number of sensors is more than that of signals. To sort synchronous FH signals in underdetermined situation, in which the number of sensors is less than that of signals, Fu and Sha used underdetermined blind source separation (UBSS) method [19,20] based on time frequency (TF) analysis. In general, in UBSS situation, the major steps to sort synchronous FH signals are estimating mixing matrix and calculating DOAs [19,20]. Sha ignored the frequency effect on mixing matrix, so the results were not useful in practice. Fu modified time frequency ratio of mixtures algorithm to estimate frequencies and mixing matrix, and then DOA was obtained to sort synchronous FH signals. However, the mixing matrix estimation in Fu’s method is not satisfactory, which leads to low estimation accuracy of DOA. Many other alternative methods are introduced, such as [21,22].

Many algorithms have been reported to deal with the UBSS problem; sparse component analysis (SCA) is an efficient method [21,22]. Based on SCA, UBSS can obtain favorable results by utilizing the sparsity of signals [21,23]. Short time Fourier transformation (STFT) and wavelet transform (WT) are usually utilized to describe sparse signal in TF domain [22,24,25,26]. Many single source point (SSP) detection methods have been proposed to improve the accuracy of mixing matrix estimation. The single source points refer to those TF vectors with only one source contribution. A time frequency ratio of mixtures (TFROM) method was proposed in [27] by calculating the ratio values to estimate mixing matrix at each TF point. This method requires that only one source is active at TF points so that the imaginary parts of the STFT coefficients are equal to zero. If more sources are active, the coefficients have complex values. A new method to detect SSP was introduced in [28], which calculated absolute directions of real parts and imaginary parts of TF coefficients. If the absolute directions of TF points were the same, these points were considered as SSPs. Similar methods were proposed in [29,30]. In [29], the mixing matrix estimation method was improved by Kernel K-hyperline clustering technology. Sun improved the absolute directions methods in [25] by ignoring low-energy points in TF domain. Xie modified the SSPs detection method in [31], which utilized the assumption that the trace of Wigner–Ville distribution (WVD) was equal to its unique nonzero eigenvalue. The methods based on detecting auto term TF points were adopted in [32,33,34] to estimate mixing matrix with the same assumption in [31]. However, these SSP detection methods [27,28,29,30,31] and the auto term TF point detection methods [32,33,34] are only suitable for real value mixing matrix. Since the mixing matrix of FH signals is complex-valued, some methods that deal with complex-valued mixing matrix estimation are discussed. Li and Nie considered the complex-valued mixing matrix estimation problem in [35,36] based on SSPs detection. Li introduced that the normalizations of coefficients of the Vandermonde mixing matrix were equal to one and the corresponding mixing coefficients could be calculated by utilizing a matrix inverse operation, so the mixing matrix estimation could be estimated by k-means clustering algorithm [35]. A similar theorem was introduced based on the inherent property of the Vandermonde mixing matrix in [36]. Although complex-valued mixing matrix estimation methods are developed, the performance is not satisfactory. An improved SSPs detection method is proposed in this paper. To recover sources, the methods in [37,38,39] based on subspace projection assumed that the number of active sources at any TF point was no more than the number of sensors, thus sources could be calculated by inverse STFT. Practically, in the subspace projection method, since these methods do not consider the effect of the residual sources, a threshold value is chosen to calculate the residual power in this paper.

Firstly, a new criterion is proposed to identify SSPs to improve mixing matrix estimation performance. Secondly, another improved source recovery method is introduced to estimate frequencies according to subspace projection. With the greatly improved estimations of mixing matrix and frequencies, the DOA of each piece of FH signals can be calculated. Finally, synchronous orthogonal FH signals can be sorted in underdetermined condition.

This paper is organized as follows. In Section 2, the FH signal model is introduced. The proposed methods are introduced in Section 3. In Section 4, the analysis and the performance of the proposed algorithms are provided and conclusions are given in Section 5.

## 2. The Problem Formulation

### 2.1. The Signal Model

**Figure 1:**
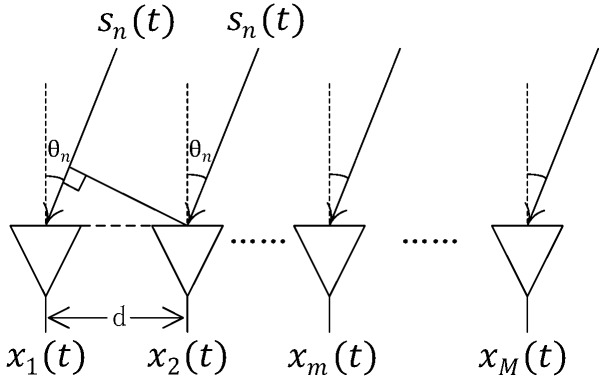
The geometry of ULA with M antennas.

Let us consider N FH signals impinging on a uniform linear array (ULA) with M antennas. The model is shown in Figure 1.

The nth FH signal is written as

(1) $$s_{n}(t)=b_{n}e^{j(2\pi f_{n}(t)t+\varphi_{n}(t))},\;0<t\leq T,$$

where $b_{n}$ is the nth signal amplitude, and $f_{n}(t)$ is the FH hopping instantaneous frequency. Phase $\varphi_{n}(t)$ is affected by frequency and modulation. T is the observation time. To avoid interference of irrelevant aspects, the amplitude and the phase can be ignored in this paper. The delay time cannot be ignored when signals impinge on different antennas. The propagation time-delay from the mth antenna to the first antenna of the nth source can be formulated as follow according to Figure 1,

(2) $$\tau_{m,n}=\frac{1}{c}(m-1)d\cos\theta_{n},\;m=1,\ldots ,M,\;n=1,\ldots ,N,$$

where c denotes the speed of light, d denotes the element space and $\theta_{n}$ denotes the nth direction of arrival (DOA). Therefore, the mth received signals can be written as

(3) $$\begin{array}{ll} x_{m}(t) & =\sum_{n=1}^{N}s_{n}(t-\tau_{m,n})+v_{m}(t)\;\;\\ & =\sum_{n=1}^{N}s_{n}\left(t\right)e^{-j2\pi f_{n}\left(t\right)\tau_{m,n}},\;m=1,\ldots ,M, \end{array}$$

where $v_{m}(t)$ is the noise. The vector formulation of (3) is

(4) x(t)=A(t)s(t)+v(t), 0<t≤T,

where $x(t)={\left[x_{1}(t),\ldots ,x_{M}(t)\right]}^{T}$, $s(t)={\left[s_{1}(t),\ldots ,s_{N}(t)\right]}^{T}$, and $v(t)={\left[v_{1}(t),\ldots ,v_{M}(t)\right]}^{T}$. Assume that the FH signals are not hopping in delay time, thus, according to (1) and (2), the transmission coefficient matrix A(t) is written as

(5) $$A(t)=\left[\begin{array}{cccc} e^{j\Omega_{1,1}(t)} & e^{j\Omega_{1,2}(t)} & \cdots & e^{j\Omega_{1,N}(t)}\\ e^{j\Omega_{2,1}(t)} & e^{j\Omega_{2，2}(t)} & \cdots & e^{j\Omega_{2,N}(t)}\\ \vdots & \vdots & \ddots & \vdots \\ e^{j\Omega_{M,1}(t)} & e^{j\Omega_{M,2}(t)} & \cdots & e^{j\Omega_{M,N}(t)} \end{array}\right]\;\;\;,$$

where $\Omega_{m,n}(t)=-2\pi f_{n}(t)\tau_{m,n}$. Obviously, according to (2), A(t) is a Vandermonde matrix. The data model in (4) is the same with blind source separation, so A(t) is the mixing matrix. The mixing matrix of FH signals changes with hopping frequencies and it is not easy to be estimated. In this paper, firstly the hop timings are obtained by other methods [40,41]. Then, the mixing matrix is constant in hop duration. The data model can be written in the kth hop duration

(6) $$x(t)=As(t)+v(t)\;=\sum_{n=1}^{N}a_{n}s_{n}(t),\;t_{k-1}<t\leq t_{k},$$

where $a_{n}$ is the nth column of the mixing matrix. To identify FH signal slices, more parameters need to be estimated, such as DOAs and frequencies, which are included in the mixing matrix. Thus, the major task is to estimate the mixing matrix.

### 2.2. The UBSS Assumptions

Under UBSS condition, the number of sensors is less than the number of sources. Similar to other UBSS methods [19,20], the following two assumptions should be satisfied. First, any M × M sub-matrix of A is full rank so that all sources can be recovered. This assumption is easily satisfied due to the Vandermonde structure of mixing matrix. Second, suppose that only one source is active at some points in TF domain. FH signal is sparse in time-frequency domain. Single-source-points detection method is effective to deal with sparse signals. The method in [42] assumes that only one source is active at each time-frequency point. The algorithm in [27] requires that there exists at least one time-frequency point of single-source-occupancy for each source. The algorithms in References [35,36,43] assume that there exist some points in the TF domain where only a single source is active and such points must exist for each source. Therefore, the second assumption is reasonable. If the frequency hopping signal does not collide, this assumption is valid.

## 3. The Proposed Algorithms

In this section, firstly, the proposed SSPs detection algorithm is introduced to estimate mixing matrix. Secondly, the improved subspace projection algorithm is proposed to recover sources.

### 3.1. Single Source Detection

For simplicity, STFT is applied on both side of (6) without noise term. The mixtures in TF domain can be written as

(7) $$X\left(t,f\right)=AS\left(t,f\right)=\sum_{n=1}^{N}a_{n}S_{n}\left(t,f\right),$$

where $X\left(t,f\right)={\left[X_{1}\left(t,f\right),\ldots ,X_{M}\left(t,f\right)\right]}^{T}$ are STFT coefficients of received signals, and $S\left(t,f\right)={\left[S_{1}\left(t,f\right),\ldots ,S_{N}\left(t,f\right)\right]}^{T}$ are the STFT coefficients of sources.

Since the methods in [35,36] treat a large number of multi-source points as single-source points, the estimation results of mixing matrix are not satisfactory. Therefore, a new theorem is proposed to detect the single source points in TF domain.

**Theorem** **1.** *At any TF point* $\left(t_{a},f_{a}\right)$ *, if only one source* $s_{n}\left(t\right)$ *contributes its power, the following condition is required*:

(8) $$\sum_{p=1}^{M-1}\sum_{q=p+1}^{M}\left|\frac{\left|X_{q}\left(t_{a},f_{a}\right)\right|}{\left|X_{p}\left(t_{a},f_{a}\right)\right|}-1\right|<\varepsilon ,$$

*where 0<ε≪1. ε is an empirical value and it depends on the signal ratio to noise (SNR). The effect of ε on estimation accuracy is discussed in Section 4.*

**Proof:**  The proof is given in Appendix A. ☐

### 3.2. The Mixing Matrix Estimation

After detecting all SSPs, the mixing matrix can be estimated by clustering methods. Suppose that $G_{n}$ is a TF region which contains all SSPs of $s_{n}\left(t\right)$. If the second row of the mixing matrix can be obtained, the mixing matrix is obviously calculated based on the Vandermonde structure. According to (7),

(9) $$\frac{X_{2}\left(t_{a},f_{a}\right)}{X_{1}\left(t_{a},f_{a}\right)}=\frac{a_{2,n}S_{n}\left(t_{a},f_{a}\right)}{a_{1,n}S_{n}\left(t_{a},f_{a}\right)},\;\left(t_{a},f_{a}\right)\in G_{n},$$

where $a_{i,n}$ represents the ith row of the nth column of mixing matrix. Because the first row elements of Vandermonde matrix are equal to one, the following result can be obtained

(10) $$a_{2,n}=\frac{X_{2}\left(t_{a},f_{a}\right)}{X_{1}\left(t_{a},f_{a}\right)},\;\left(t_{a},f_{a}\right)\in G_{n}.$$

To improve the mixing matrix estimation accuracy, based on (5) and (9), $\Omega_{2,n}$ which corresponds to the SSP $\left(t_{a},f_{a}\right)\in G_{n}$, is represented as

(11) Ω2,n={arctanIm{a2,n}Re{a2,n}+πif Re{a2,n}<0 and Im{a2,n}Re{a2,n}<0arctanIm{a2,n}Re{a2,n}others.

Cluster $\Omega_{2,n}$ into N different classes. The proposed algorithm utilizes the hierarchical clustering technique to estimate clustering centers $\left\{{\hat{\Omega }}_{2,n},\;n=1,\ldots ,N\right\}$. Although it may not be the best clustering algorithm, the clustering method will be studied in the future work.

Therefore, the mixing matrix estimation is calculated by

(12) $$\begin{array}{l} {\hat{a}}_{2,n}=\exp\left(j{\hat{\Omega }}_{2,n}\right)\;n=1,\ldots ,N\\ \hat{A}=\left[\begin{array}{ccc} 1 & \cdots & 1\\ {\hat{a}}_{2,1} & \cdots & {\hat{a}}_{2,N}\\ \vdots & \ddots & \vdots \\ {\hat{a}}_{2,1}^{M-1} & \cdots & {\hat{a}}_{2,N}^{M-1} \end{array}\right] \end{array}$$

### 3.3. FH Signals Recovery

An improved subspace projection method is proposed which is inspired by the method in [38]. The method in [38] assumed that there were no more than M active sources at each TF point. It calculated the minimum value of the product of the orthogonal projection matrix of mixing matrix and the TF domain mixtures to identify the sources. However, this method did not consider the effect of redundant signals. That is why the number of active sources at each TF point is always estimated as M. To improve the accuracy of the recovered FH signals, the proposed method in this paper sets a threshold to solve this problem. Suppose that there exist l (l≤M) FH signals at each TF point and l is a variable number. The corresponding mixing matrix column vectors are denoted by $\left[a_{k_{1}},\ldots ,a_{k_{l}}\right]$, where, $\left\{k_{1},\ldots ,k_{l}\right\}\in \left\{1,\ldots ,N\right\}$, $k_{1}<k_{2}<\cdots <k_{l}$. The l FH signals can be identified by

(13) $$\begin{array}{c} {}[a_{k_{1}},\ldots ,a_{k_{l}}]=\arg\;\underset{l}{\min}\left\{{\left\|Q_{l}X(t,f)\right\|}_{2}<\varepsilon_{0}{\left\|X(t,f)\right\|}_{2}\right\}\\ {\hat{S}}_{l}(t,f)={\left(A_{l}^{H}A_{l}\right)}^{-1}A_{l}X(t,f) \end{array}\;\;\;,$$

where $Q_{l}=Ι-A_{l}{\left(A_{l}^{H}A_{l}\right)}^{-1}A_{l}$, and $A_{l}=\left[a_{k_{1}},\ldots .,a_{k_{l}}\right]$. $\varepsilon_{0}$ is a small value and it is relative to redundant weak signals. The recovered FH sources in time domain can be estimated by inverse STFT. The frequencies $\left\{{\hat{f}}_{n},\;n=1,\cdots ,N\right\}$ can be calculated by fast Fourier transformation (FFT).

### 3.4. DOA Estimation and Splicing of FH Signals

For synchronous FH signals, hopping times of FH signals are the same. To splice FH signals, DOAs are required. According to the mixing matrix in (5), DOAs of FH signals in kth hop duration can be estimated by

(14) $${\hat{\theta }}_{n}=\arccos\frac{-{\hat{\Omega }}_{2,n}c}{2\pi {\hat{f}}_{n}d}.$$

DOAs do not change in a short time. If the DOAs of segments are approximately equal, these segments belong to the same FH signal. Therefore, comparing the DOAs between hop durations is an effective way to splice FH signals. The decision criterion is

(15) $$\left|{\mathrm{DOA}}_{i}-{\mathrm{DOA}}_{j}\right|\begin{array}{c} \overset{H_{1}}{>}\\ \underset{H_{0}}{<} \end{array}r,$$

where ${\mathrm{DOA}}_{i}$ and ${\mathrm{DOA}}_{j}$ represent different segments, stands for the hypothesis of “not the same FH signals segments”, $H_{0}$ stands for “the same FH signals segments” and r is a judgment threshold. The value of r will be described in Section 4.3.

In Summary, the proposed algorithm is given in Algorithm 1.
**Algorithm 1 The proposed algorithm**Input: The received signalsStep 1: Segment the received signals based on the known or estimated hop timings.
Step 2: In one hop duration, calculate the STFT $X\left(t,f\right)=\left\{X_{1}\left(t,f\right),\ldots ,X_{M}\left(t,f\right)\right\}$ of the received signals. For each TF point $\left(t_{a},f_{a}\right)$, calculate $\sum_{p=1}^{M-1}\sum_{q=p+1}^{M}\left|\frac{\left|X_{q}\left(t_{a},f_{a}\right)\right|}{\left|X_{p}\left(t_{a},f_{a}\right)\right|}-1\right|$ and utilize Theorem (8) to detect signal-source-points.
Step 3: Calculate $\frac{X_{1}\left(t_{a},f_{a}\right)}{X_{1}\left(t_{a},f_{a}\right)}$ for all SSPs. Based on (11), $\Omega_{2,n}$ can be obtained and ${\hat{\Omega }}_{2,n}$ is calculated via clustering method.
Step 4: Calculate the mixing matrix by (12).
Step 5: Estimate $\hat{S}(t,f)$ by (13), and calculate the inverse STFT to obtain FH signals. The frequency $\left\{\hat{f},\;n=1,\ldots ,\;N\right\}$ can be calculated by FFT.
Step 6: Calculate DOAs $\left\{{\hat{\theta }}_{n},\;n=1,\ldots ,N\right\}$ by (14).
Step 7: In another hop duration, repeat Step 2 to Step 6.
Step 8: Compare the DOAs in different hop durations. The recovered FH signals can be spliced according to (15).
Output: $\left\{{\hat{\theta }}_{n},\;n=1,\ldots ,N\right\}$, $\left\{{\hat{f}}_{n},\;n=1,\ldots ,N\right\}$ and recovered FH signals.

## 4. Simulations

In this section, numerical results are given to demonstrate the effectiveness of the proposed algorithms. In all experiments, M=4. Suppose sample size L=256 and N=5. The synchronous FH signals parameters are shown in Table 1. In this section, the thresholds ε, $\varepsilon_{0}$ and r are set to 0.2, 0.1 and 2, respectively. The influence of thresholds on the estimation performance is discussed later.

Hop timings can be estimated by the methods in [40,41]. This parameter is ignored in Table 1. In the following simulations, the SNR is written as

(16) $$\mathrm{SNR}=10\log_{10}(\frac{{\left\|s(t)\right\|}_{2}^{2}}{N\sigma^{2}}).$$

Following [44], the performance of parameter estimation can be measured by Root mean square error (RMSE) which is defined as

(17) $$\mathrm{RMSE}=\sqrt{\frac{\sum_{n=1}^{N}{\left(\eta_{n}-{\hat{\eta }}_{n}\right)}^{2}}{N}},$$

where $\eta_{n}$ is the nth original parameter, and ${\hat{\eta }}_{n}$ is the nth estimation value.

### 4.1. Mixing Matrix Estimation

The performance of the mixing matrix estimation is demonstrated by mean square error (MSE) which is defined as

(18) $$\mathrm{MSE}=10\log_{10}\left(\frac{{\left\|A-\hat{A}\right\|}_{2}^{2}}{N}\right).$$

A small MSE indicates superior quality. In addition, the columns of the estimated mixing matrix are normalized and the uncertainty of the columns is removed.

**Figure 2:**
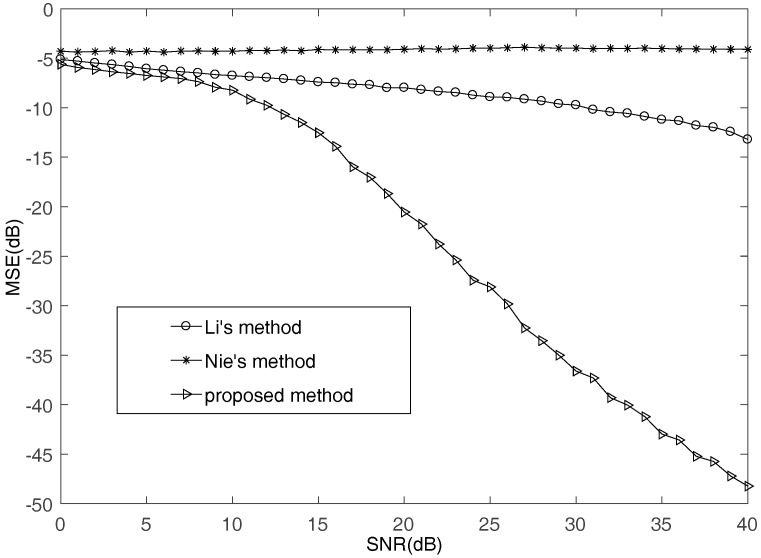
The MSE comparisons of different methods.

First, the underdetermined situation where M=4 and N=5 is considered. This experiment does 1000 Monte Carlo tests. Figure 2 illustrates the MSE performance of the proposed method comparing with Li’s method in [35] and Nie’s method in [36] with SNR increasing. It can be seen that the proposed method can obtain higher accuracy than other algorithms. Since the methods of Li and Nie treat a large number of multi-source points as single-source points, the estimation results of mixing matrix are not satisfactory.

**Figure 3:**
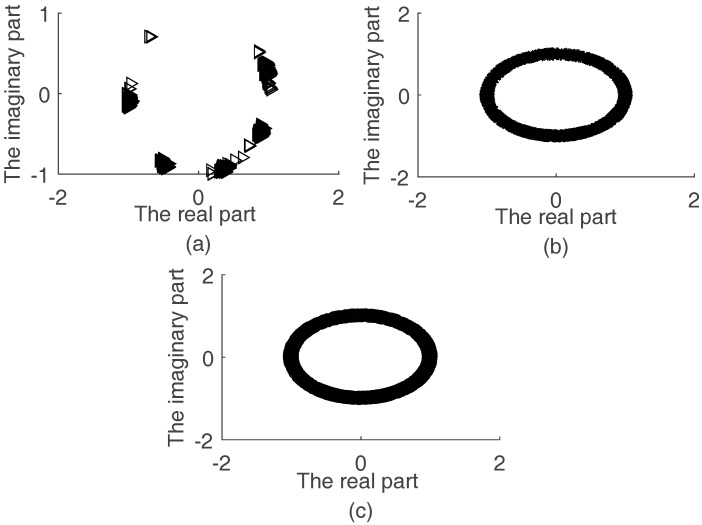
The scatter diagram of $\left\{a_{2,n},\;n=1,\cdots ,N\right\}$ for SNR=15 dB: (**a**) the scatters of the proposed method; (**b**) the scatters of Nie’s method; and (**c**) the scatters of Li’s method.

Figure 3a shows the scatter diagrams of real parts and imaginary parts of $\left\{a_{2,n},\;n=1,\cdots ,N\right\}$ which are calculated by (10) when SNR=15 dB. Figure 3a shows N clusters clearly. However, it is hard to identify clusters from Figure 3b,c, which are calculated by Nie’s and Li’s methods, respectively. Each cluster represents $a_{2,n},\;n=1,\ldots ,\;N$. Because the proposed method detects fewer error points than the other two methods, the proposed method works very well.

**Figure 4:**
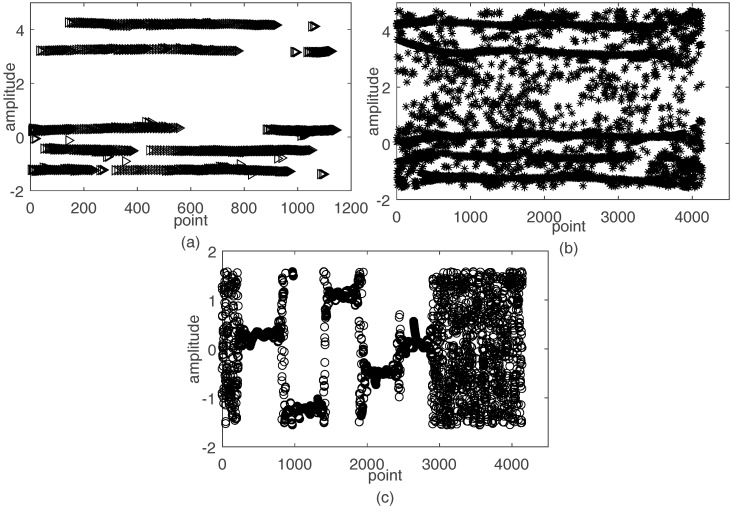
The amplitude of $\left\{\Omega_{2,n},\;n=1,\cdots ,N\right\}$ for SNR=15 dB: (**a**) the performance of the proposed method; (**b**) the performance of Nie’s method; and (**c**) the performance of Li’s method.

$\left\{\Omega_{2,n},\;n=1,\cdots ,N\right\}$ calculated by (11) are shown in Figure 4a when SNR=15 dB. The amplitudes represent the values of $\left\{\Omega_{2,n},\;n=1,\cdots ,N\right\}$. Since the proposed method has eliminated many unreliable SSPs, the $\left\{\Omega_{2,n},\;n=1,\cdots ,N\right\}$ in Figure 4a clearly show N straight lines, which represent the clusters. Although, Nie’s method and Li’s method can also show straight lines in Figure 4b,c, respectively, many disturbing points that lead to the bad results of the mixing matrix exist.

**Figure 5:**
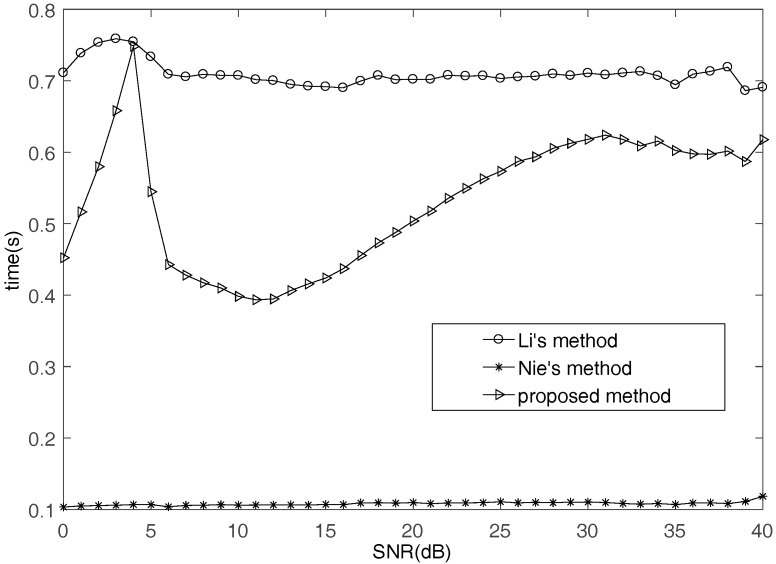
CPU time comparison of different methods.

To analyze the complexity of these methods, CPU time is calculated. Our simulations are performed in MATLAB R2009a using an Inter Core I3-2100 +310 GHz processor. The operating system is Microsoft Windows XP and the memory is 2 GB. The average CPU time (s) for Monte Carlo tests against SNR levels is shown in Figure 5. The proposed method utilizes all mixtures in TF domain to detect SSPs, while Nie’s method just uses two mixtures. That is why the proposed method cost more time than Nie’s method. Because the numbers of SSPs are different in different SNR, the cost time of proposed method is not the same. The cost time of the proposed method is increasing with the number of SSPs.

**Figure 6:**
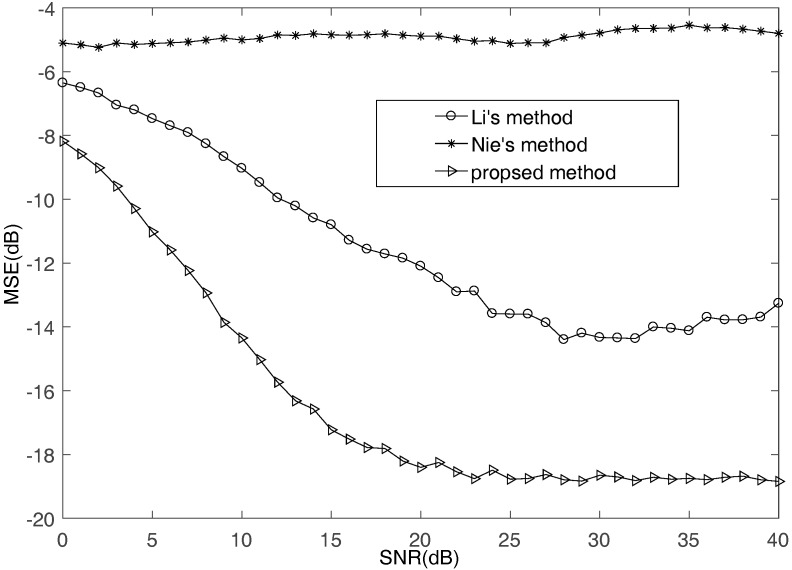
The MSE comparison of different methods for M=4 and N=6.

Second, to analyze the source number effect on MSE performance, the underdetermined situation where M=4 and N=6 is considered. The comparison of the MSE for M=4 and N=6 is shown in Figure 6. It demonstrates that the performance of the proposed method is also better than the others when the source number increases to six. Comparing with Figure 2, it can be seen that the proposed method gets large MSE when N=6. In other words, the performance becomes bad when the number of sources increases. Therefore, the number of FH signals cannot be much larger than that of antennas.

**Figure 7:**
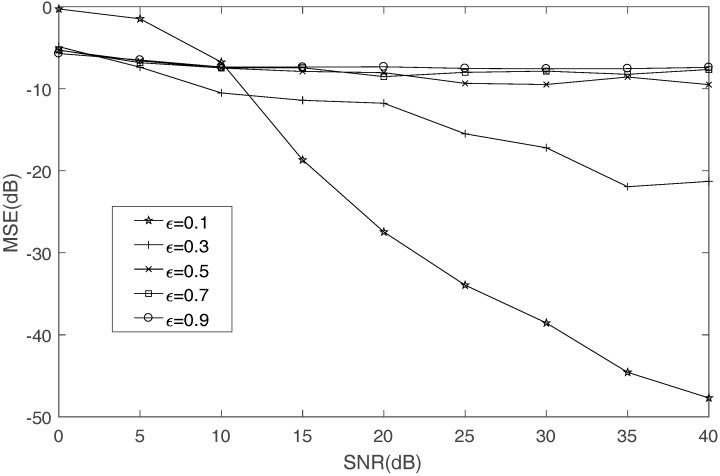
The effect of ε on mixing matrix.

Third, the influence of threshold ε on the MSE of mixing matrix is discussed. When ε is too small, not enough single-source-points exist to be used for estimating mixing matrix in low SNR. This will lead to wrong results. When ε is too large, many more multi-source points exist to be used for estimating mixing matrix. This will also lead to wrong results. The effect of ε is shown in Figure 7. When ε=0.1, the performance is bad in low SNR. When ε is greater than 0.3, the results are not credible. Through the above analysis, it is reasonable to set ε=0.2.

**Figure 8:**
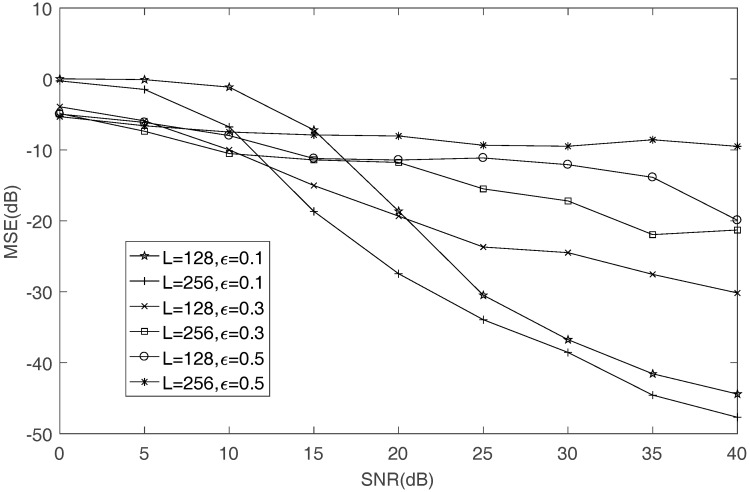
The effect of sample size on the MSE of mixing matrix estimation.

The effect of sample size on the performance of mixing matrix is discussed in this part. Figure 8 shows that, when L=256, more accuracy can be obtained than L=128 if ε=0.1. However, if ε is chosen too large, the accuracy is worse than L=128. Thus, in the case of large sample size, such as L=256, a small threshold ε should be chosen, which leads to higher accuracy.

**Figure 9:**
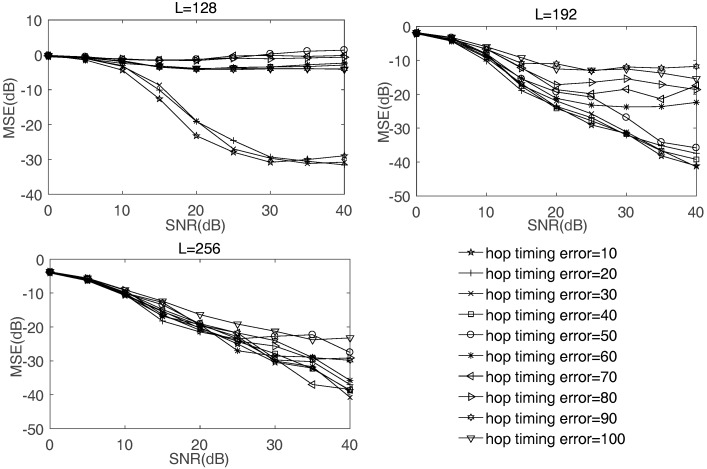
The impact of hop timing errors on the MSE of mixing matrix estimation.

Fourth, the influence of hop timing error on the MSE of mixing matrix is discussed. Figure 9 shows the impact of different hop timing errors on different sample sizes. When sample size is L=128, the performance of mixing matrix estimation becomes bad when hop timing error is greater than 30 samples. When sample size is L=192, the performance of mixing matrix estimation becomes bad when hop timing error is greater than 60 samples. When sample size is L=256, the performance of mixing matrix estimation is not bad even if hop timing error is greater than 100 samples. Thus, the greater the radio of the hop timing error to sample size is, the worse the result is. According to the existing hop timing estimation algorithms in [40,41], the hop timing can be estimated correctly in a certain range of error. Hop timing error is not very restricted to the proposed method.

### 4.2. FH Signals Recovery

The recovery performance is quantified by source to interference ratio (SIR), which is defined as

(19) $$\mathrm{SIR}=10\log_{10}\left(\frac{\sum_{n=1}^{N}Ε\left[s_{n}^{2}\left(t\right)\right]}{\sum_{n=1}^{N}Ε\left[{\left(s_{n}\left(t\right)-{\hat{s}}_{n}\left(t\right)\right)}^{2}\right]}\right),$$

where ${\hat{s}}_{n}\left(t\right)$ is the nth recovered signal. A large SIR indicates superior quality.

**Figure 10:**
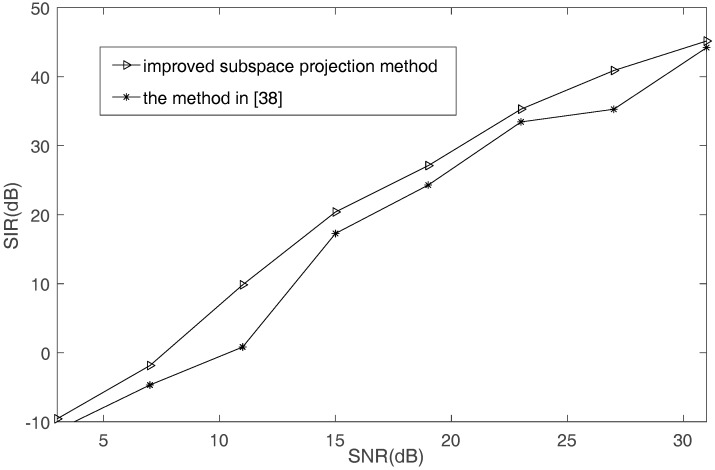
The SIR comparison of different recovered methods.

First, Figure 10 compares the recovery performance between the improved subspace projection method and the method in [38]. These two methods utilize the mixing matrix that is estimated by the proposed SSPs detection method. It can be seen that the improved subspace projection method performs better than the method in [38].

**Figure 11:**
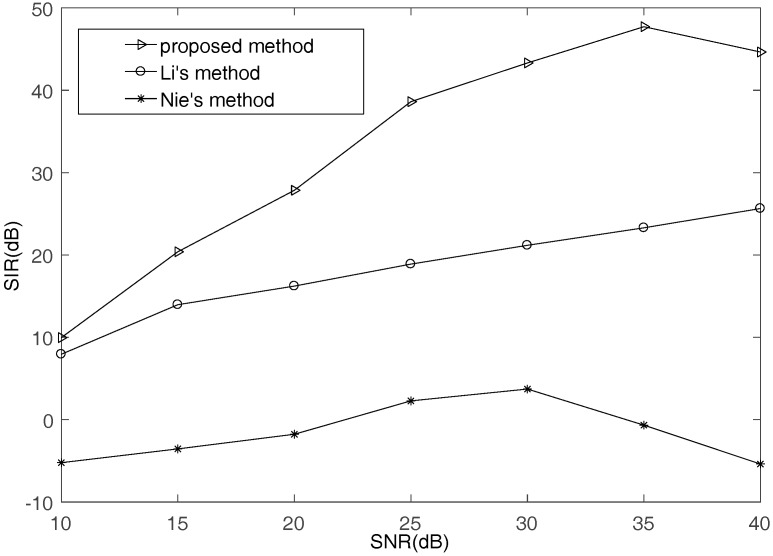
The SIR comparison of different mixing matrix estimation methods.

Second, according to estimated mixing matrices that are obtained by the three methods, the improved subspace projection method is used to recover signals, and the performance is shown in Figure 11. It can be known that the higher the estimation accuracy of the mixing matrix is, the better the recovered performance is. This also proves that the proposed SSPs detection method is effective and can improve recovered signal performance.

**Figure 12:**
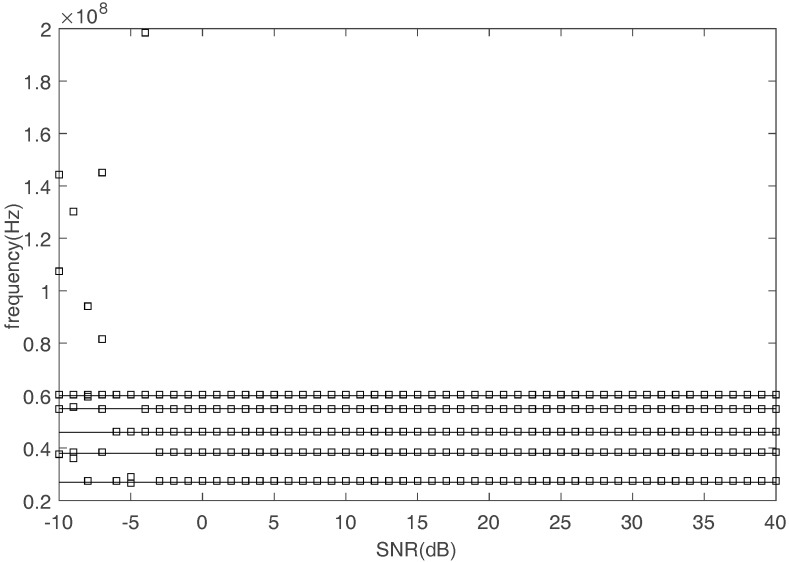
The comparison between the true and estimated frequency.

**Figure 13:**
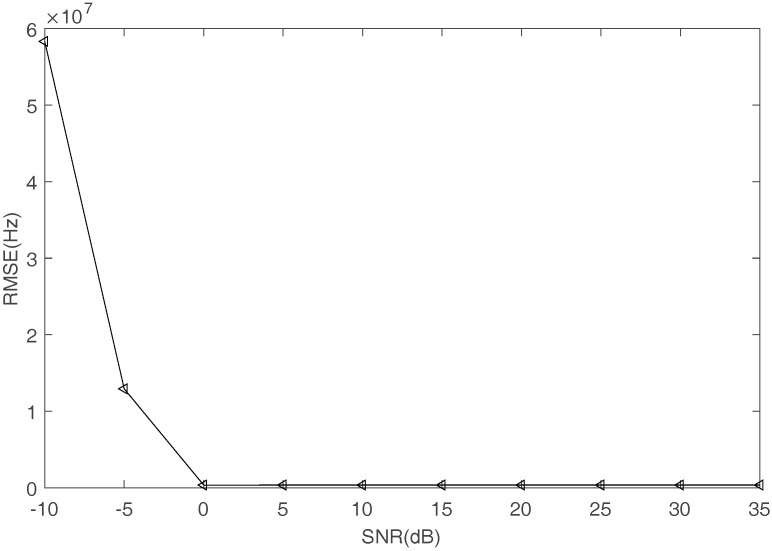
The RMSE of frequency estimation.

Third, frequencies can be estimated by FFT operator when the signals are obtained by the proposed recovery method in time domain. Figure 12 shows the comparison between the true and the estimated frequencies with SNR increasing. The square represents the estimated frequency and the straight line represents the true frequency. Figure 13 shows the RMSE of frequency estimation. Even when SNR is equal to 0 dB, the proposed methods can estimate the frequencies accurately.

**Figure 14:**
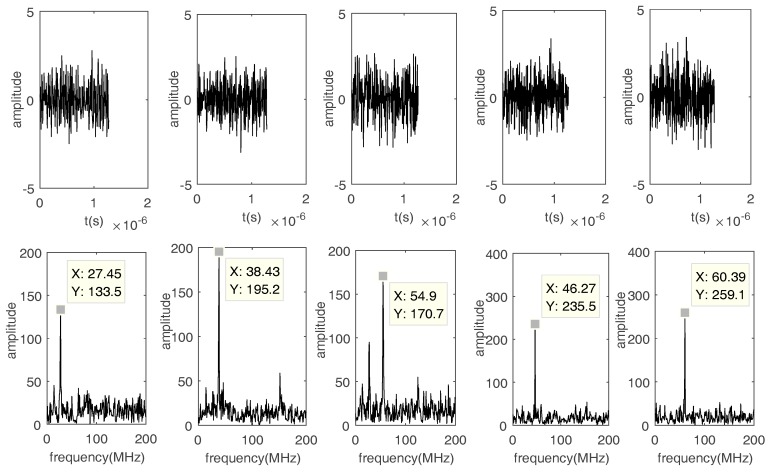
The waveforms and FFT results when SNR = 0 dB and $\varepsilon_{0}$ = 0.1.

**Figure 15:**
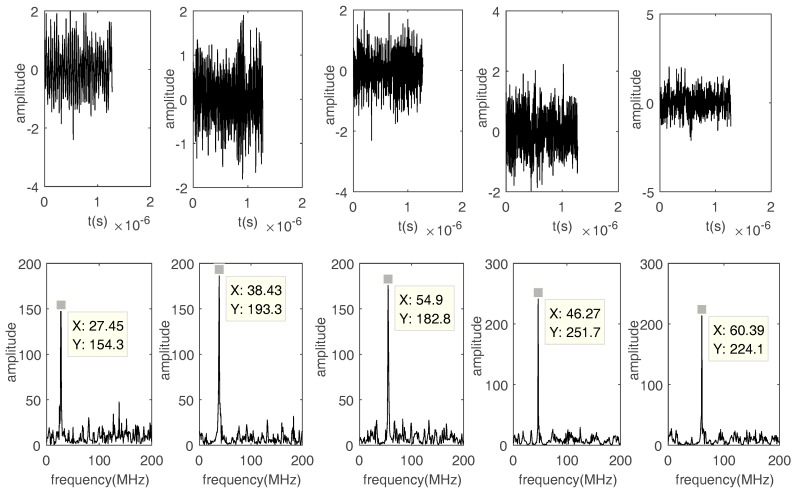
The waveforms and FFT results when SNR = 0 dB and $\varepsilon_{0}$ = 0.5.

Fourth, the influence of the threshold $\varepsilon_{0}$ on the SIR of the recovered FH signals is discussed. $\varepsilon_{0}$ effects the performance of waveform recovery. Figure 14 and Figure 15 show that frequencies can be calculated by FFT even when using bad recovered waveforms. Thus, if the mixing matrix and frequencies are calculated, $\varepsilon_{0}$ has a minimal impact on the performance of DOA estimation.

### 4.3. DOA Estimation and Splicing FH Signals

**Figure 16:**
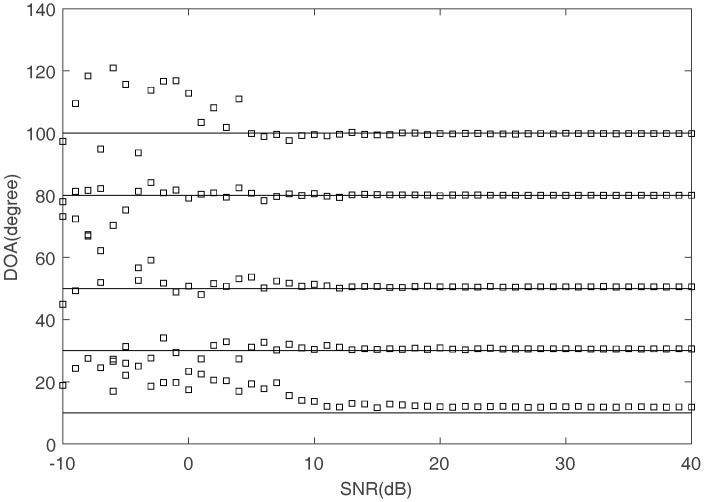
The comparison between the true and estimated DOA.

**Figure 17:**
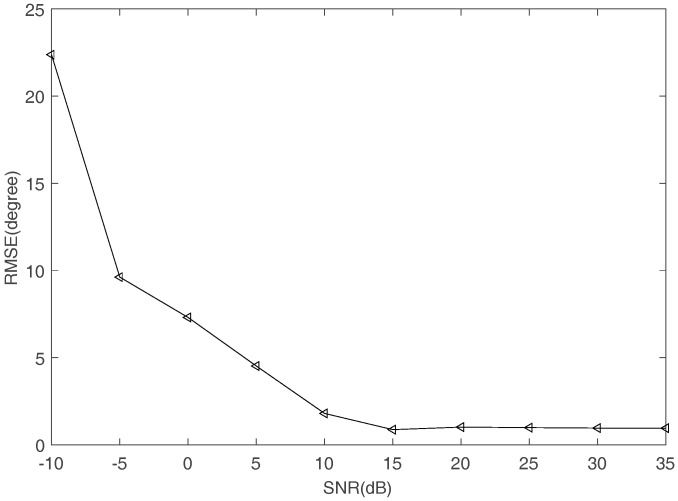
The RMSE of DOA estimation.

The DOAs estimation can be obtained by (14) using the estimated mixing matrix and the frequency which are obtained by the proposed methods. Figure 16 shows the comparison of the true DOA and the estimated DOA with SNR increasing. The square represents the estimated value of DOA and the straight line means the true value of DOA. Figure 17 shows the RMSE of DOA estimation. It can be seen that the estimated DOAs are almost the same as the true ones when SNR is higher than 10 dB.

To sort FH signals, DOAs of different hop durations are estimated. Table 2 lists the RMSE performance of two hop durations when SNR is changing. Comparing the data in Table 2 with the data in Table 1, the DOA estimation is close to the true value when SNR is increasing. It can be seen that the RMSE of DOA estimation remains less than 1° when SNR is higher than 15 dB. In addition, the proposed algorithms can successfully sort synchronous orthogonal FH signals in the underdetermined case. The correlation of mixing matrix columns is related to the product of DOA and frequency. Under the assumption that any M×M sub-matrix of mixing matrix has full rank, the mixing matrix can be estimated even if DOA of FH signals are the same. Therefore, r can be selected based on the RMES of DOA estimation. r should be two times larger than that of RMSE of DOA estimation. According to Figure 17, the threshold r in (15) can be set to 2° so that different hops can be spliced.

## 5. Conclusions

This paper proposes a novel method to detect SSPs in TF domain and also an improved subspace projection method is proposed to recover signals. The synchronous FH sorting problem is solved in UBSS situation. The proposed SSPs detection method improves the complex-valued mixing matrix estimation accuracy. In this paper, DOAs and frequencies are also estimated by the improved subspace projection method. The RMSE of DOA estimation based on the proposed methods is less than 1° when SNR is higher than 15 dB in the underdetermined case. The task of sorting FH signals has been completed by the proposed methods. We will try to improve DOA estimation accuracy in low SNR in the future work.

## Figures and Tables

**Table 1:** The Synchronous FH signals parameters.

Signals	Frequency (MHz)	DOA(°)
Signal 1	27, 33	100
Signal 2	38, 44	50
Signal 3	55, 61	80
Signal 4	46, 52	30
Signal 5	60, 66	10

**Table 2:** The RMSE performance of the proposed algorithms.

		SNR = 15 dB	SNR = 25 dB	SNR = 30 dB	SNR = 40 dB
Hop duration 1	Estimation DOAs (°)	99.4272	99.7098	99.9485	99.8157
		50.6795	50.4218	50.5310	50.5380
		80.1626	80.0028	79.9600	79.9867
		30.3958	30.5444	30.6250	30.5645
		11.6847	12.0813	11.9910	11.8942
	RMSE	0.8741	0.9890	0.9634	0.9197
Hop duration 2	Estimation DOAs (°)	99.9952	99.9788	100.0327	100.0226
		49.8621	49.9086	49.8987	49.8865
		79.6136	80.0633	49.8987	49.8865
		31.7015	31.2438	30.9527	31.0309
		10.3528	9.6034	9.1250	9.2847
	RMSE	0.7985	0.5860	0.5805	0.5626

## Notation

$A^{T}$, $A^{-1}$, $A^{H}$	the transpose, inverse and conjugate transpose of A, respectively.
‖A‖	$l_{2}$ norm of matrix A
|a|	the absolute value of a
Re{a}	the real part of a
Im{a}	the imaginary part of a

## Appendix A

This part shows the proof of theorem.

Suppose $\left(t_{a},f_{a}\right)$ is an SSP of $s_{n}\left(t\right)$, the (7) can be written as

(A1) $$X\left(t_{a},f_{a}\right)=a_{n}S_{n}\left(t_{a},f_{a}\right)\;\;.$$

At the $\left(t_{a},f_{a}\right)$ TF point, the STFT of mth mixture is

(A2) $$X_{m}\left(t_{a},f_{a}\right)=a_{m,n}S_{n}\left(t_{a},f_{a}\right)\;\;,$$

where $a_{m,n}$ is the coefficient of mixing matrix. Expand the real and the imaginary part of (A2)

(A3) Re{Xm(ta,fa)}+jIm{Xm(ta,fa)}   =(Re{am,n}+jIm{am,n})(Re{Sn(ta,fa)}+jIm{Sn(ta,fa)}).

Then, it can be obtained that

(A4) Re{Xm(ta,fa)}=Re{am,n}Re{Sn(ta,fa)}−Im{am,n}Im{Sn(ta,fa)}Im{Xm(ta,fa)}=Im{am,n}Re{Sn(ta,fa)}+Re{am,n}Im{Sn(ta,fa)}.

Calculate the square of Re{Xm(ta,fa)} and Im{Xm(ta,fa)}, and the following formulation can be obtained

(A5) Re{Xm(ta,fa)}2+Im{Xm(ta,fa)}2=[Re{am,n}2+Im{am,n}2]Re{Sn(ta,fa)}2+[Re{am,n}2+Im{am,n}2]Im{Sn(ta,fa)}2.

Based on the structure of mixing matrix in (5), Re{am,n}2+Im{am,n}2=1. Then, (A5) is written as

(A6) Re{Xm(ta,fa)}2+Im{Xm(ta,fa)}2=Re{Sn(ta,fa)}2+Im{Sn(ta,fa)}2 .

Due to the arbitrary m, the equation as follows is obtained at the $\left(t_{a},f_{a}\right)$ point

(A7) Re{Xp(ta,fa)}2+Im{Xp(ta,fa)}2=Re{Xq(ta,fa)}2+Im{Xq(ta,fa)}2

and it is equal to

(A8) $$\left|X_{p}\left(t_{a},f_{a}\right)\right|=\left|X_{q}\left(t_{a},f_{a}\right)\right|\;.$$

It indicates that, if $\left(t_{a},f_{a}\right)$ is an SSP, the mixtures are the same absolute value in TF domain. If there is no noise, the (A8) can be rewritten as

(A9) $$\sum_{p=1}^{M-1}\sum_{q=p+1}^{M}\left|\frac{\left|X_{q}\left(t_{a},f_{a}\right)\right|}{\left|X_{p}\left(t_{a},f_{a}\right)\right|}-1\right|=0.$$

Consider the noise effect and relax the condition, a threshold ε is introduced. Then, (A9) can be written as

(A10) $$\sum_{p=1}^{M-1}\sum_{q=p+1}^{M}\left|\frac{\left|X_{q}\left(t_{a},f_{a}\right)\right|}{\left|X_{p}\left(t_{a},f_{a}\right)\right|}-1\right|<\varepsilon ,$$

where 0<ε≪1. Assume that at $\left(t_{a},f_{a}\right)$ two sources, $s_{i}\left(t\right)$ and $s_{j}\left(t\right)$, are active. Then (7) can be written as

(A11) $$X\left(t_{a},f_{a}\right)=a_{i}S_{i}\left(t_{a},f_{a}\right)+a_{j}S_{j}\left(t_{a},f_{a}\right).$$

At the TF point $\left(t_{a},f_{a}\right)$, the STFT of mth mixture is

(A12) $$X_{m}\left(t_{a},f_{a}\right)=a_{m,i}S_{i}\left(t_{a},f_{a}\right)+a_{m,j}S_{j}\left(t_{a},f_{a}\right)\;.$$

Then,

Re{Xm(ta,fa)}=Re{am,i}Re{Si(ta,fa)}−Im{am,i}Im{Si(ta,fa)}+Re{am,j}Re{Sj(ta,fa)}−Im{am,j}Im{Sj(ta,fa)}  ,Im{Xm(ta,fa)}=Im{am,i}Re{Si(ta,fa)}+Re{am,i}Im{Si(ta,fa)}+Im{am,j}Re{Sj(ta,fa)}+Re{am,j}Im{Sj(ta,fa)}  .

Since Re{am,i}2+Im{am,i}2=1 and Re{am,j}2+Im{am,j}2=1, then

(A13) Re{Xm(ta,fa)}2+Im{Xm(ta,fa)}2=Re{Si(ta,fa)}2+Im{Si(ta,fa)}2+Re{Sj(ta,fa)}2+Im{Sj(ta,fa)}2+2Re{Si(ta,fa)}Re{Sj(ta,fa)}(Im{am,i}Im{am,j}+Re{am,i}Re{am,j})+2Im{Si(ta,fa)}Im{Sj(ta,fa)}(Re{am,i}Re{am,j}+Im{am,i}Im{am,j})+2Re{Si(ta,fa)}Im{Sj(ta,fa)}(Im{am,i}Re{am,j}−Re{am,i}Im{am,j})+2Im{Si(ta,fa)}Re{Sj(ta,fa)}(Re{am,i}Im{am,j}−Im{am,i}Re{am,j})  .

Due to the Vandermonde structure of the mixing matrix, the first row coefficient of the mixing matrix $a_{1,n}$ is satisfied with Re{a1,n}=1, Im{a1,n}=0, then,

Re{X1(ta,fa)}=Re{Si(ta,fa)}+Re{Sj(ta,fa)}Im{X1(ta,fa)}=Im{Si(ta,fa)}+Im{Sj(ta,fa)} .

Calculate the square of Re{X1(ta,fa)} and Im{X1(ta,fa)}, which is shown as follows:

(A14) Re{X1(ta,fa)}2+Im{X1(ta,fa)}2=Re{Si(ta,fa)}2+Re{Sj(ta,fa)}2+Im{Si(ta,fa)}2+Im{Sj(ta,fa)}2  +2Re{Si(ta,fa)}Re{Sj(ta,fa)}+2Im{Si(ta,fa)}Im{Sj(ta,fa)} .

Assume that (A8) also holds true for MSPs; comparing (A14) and (A13), the following is obtained

(A15) Im{am,i}Im{am,j}+Re{am,i}Re{am,j}=1Im{am,i}Re{am,j}−Re{am,i}Im{am,j}=0.

Utilizing the properties Re{am,i}2+Im{am,i}2=1 and Re{am,j}2+Im{am,j}2=1, (A15) is equivalent to the following

(A16) Re{am,i}=Re{am,j}Im{am,i}=Im{am,j}.

The condition in (A16) is hash in practice. Therefore, MSPs cannot satisfy (A10).

The proof is completed.
